# Perfluorooctanoic Acid Promotes Recruitment and Exocytosis of Rodlet Cells in the Renal Hematopoietic Tissue of Common Carp

**DOI:** 10.3390/toxics11100831

**Published:** 2023-09-30

**Authors:** Maurizio Manera, Giuseppe Castaldelli, Luisa Giari

**Affiliations:** 1 Department of Biosciences, Food and Environmental Technologies, University of Teramo, St. R. Balzarini 1, 64100 Teramo, Italy; 2 Department of Environmental and Prevention Sciences, University of Ferrara, St. Borsari 46, 44121 Ferrara, Italy; giuseppe.castaldelli@unife.it (G.C.); luisa.giari@unife.it (L.G.)

**Keywords:** per- and polyfluoroalkyl substances (PFAS), kidney, toxicology, immune response, immunotoxicity, ecotoxicology, ultrastructure, chemical exposure, environmental health, aquatic organisms

## Abstract

Per- and polyfluoroalkyl substances (PFAS) are ubiquitous environmental contaminants, with perfluorooctanoic acid (PFOA) being a prominent member. PFOA poses a risk to aquatic ecosystems and human health due to its presence in water, environmental persistence, and bioaccumulation. Since rodlet cells (RCs) have emerged as potential biomarkers for chemical stressors, this study aimed to investigate the effects of sub-chronic PFOA exposure on RCs in the renal hematopoietic tissue of common carp. Three groups of fish were used: an unexposed control group and two groups exposed to environmentally relevant (200 ng L^−1^) and elevated (2 mg L^−1^) PFOA concentrations. Light and transmission electron microscopy were employed to assess RCs’ distribution patterns and exocytosis, while biometry quantified RCs in the hematopoietic tissue. The results showed that, even at environmentally relevant concentrations, PFOA significantly influenced RCs’ distribution patterns, leading to increased occurrence and cluster formation, as well as heightened exocytosis activity. This research highlights PFOA’s immunotoxicity in fish and suggests the potential of RCs as sentinel cells in the immunological response to environmental contaminants. These findings enhance our understanding of PFAS toxicity and emphasise the importance of monitoring their impact on fish as representative vertebrates and reliable animal models.

## 1. Introduction

Per- and polyfluoroalkyl substances (PFAS), commonly referred to as “forever chemicals,” constitute a class of synthetic compounds characterised by the presence of multiple fluorine atoms attached to alkyl chains. These compounds have been extensively manufactured and employed in various industrial processes and consumer products as both polymers and additives since the 1940s [1,2]. Initially, they were regarded as inert and benign, garnering little attention with respect to their environmental fate and potential health impacts [3]. However, PFAS are now widely acknowledged as pervasive global contaminants due to their extensive distribution, remarkable stability, long-lasting persistence in the environment, and potential toxicity to various organisms, including humans [3,4]. Regrettably, only a limited number of PFAS compounds are currently monitored and regulated, primarily within developed countries, which are in the process of establishing stringent guidelines [5]. While research on PFAS has expanded significantly in recent years, there remains a paucity of data concerning the effects of PFAS at environmentally relevant concentrations and the mechanisms through which PFAS can adversely impact health [3].

Among the over 4700 compounds encompassed within the PFAS class, perfluorooctanoic acid (PFOA), a fully fluorinated eight-carbon chain PFAS with a carboxylic acid end group, stands out as one of the most widespread and extensively documented [3]. PFOA is particularly prevalent in aquatic environments [6], with concentrations in surface waters ranging from levels below detection limits to hundreds of nanograms per liter, occasionally surging to microgram-per-liter levels in proximity to specific point sources, such as fluoropolymer facilities and wastewater treatment plant effluents [7,8,9]. Fish, along with other aquatic organisms, are consistently exposed to PFOA and can accumulate this compound [10,11]. Fish populations thus represent a potential avenue for human contamination through the food supply chain [12] and offer promising subjects for investigating PFOA toxicology within the context of environmental pathology [13] and the One Health approach [14].

The focus of this study is the impact of PFOA on the kidney of fish. The piscine kidney plays a pivotal role in the accumulation and excretion of PFOA [15,16], with the kidney of the common carp (*Cyprinus carpio*) serving as an excellent model for exploring various toxicological aspects, including nephrotoxicity, immunotoxicity, and endocrine disruption [17,18,19]. The carp kidney is composed of different histological elements, including nephrons, hematopoietic tissue, and thyroid follicles [20].

PFOA has been demonstrated to induce various adverse effects in fish, including oxidative stress, peroxisome proliferation, disturbances in lipid metabolism, reproductive disruption, teratological effects, and endocrine disruption, particularly affecting the thyroid and gonads with estrogenic or anti-estrogenic effects [10,21,22,23,24,25,26]. These detrimental effects can potentially impact metabolic functions in fish larvae and adults, including energy production, fecundity, growth, and development [27,28,29,30]. Furthermore, PFOA has been designated as a “presumed immune hazard to humans” by the National Toxicology Program, based on data collected from both experimental animal models and humans [31,32]. Although PFOA-induced immune system damage has been extensively studied in mammals, limited information exists regarding the immunotoxicity of PFOA in aquatic organisms, especially concerning innate immunity [16,33]. Zhang et al. (2021) [16] observed altered secretion of cytokines and antibodies in zebrafish exposed to PFOA, suggesting potential immune disorders. Additionally, an inflammatory response induced by PFOA, characterised by modified expression of certain immune-related amino acids, has been reported in zebrafish embryos and adults [30]. The study by Pecquet et al. (2020) [33] demonstrated that PFOA can impair the innate immune system in developing zebrafish embryos by reducing neutrophil migration.

Rodlet cells (RCs), exclusive to teleosts, were first described by Thélohan in 1892. Subsequently, two comprehensive reviews have focused on elucidating the nature, origin, structure, and function of these peculiar cells, highlighting their potential utility as biomarkers of exposure to chemical stressors [34,35]. RCs are primarily located in the epithelia of various organs in both freshwater and marine fish species [35,36]. Morphologically, mature RCs exhibit a thick fibrillar layer, prominent rod-shaped inclusions called rodlets, and a basally located nucleus [34,35].

Recent decades have witnessed mounting evidence suggesting that RCs are integral components of the fish innate immune system, capable of responding to various forms of tissue injury [17,35,37,38,39,40,41]. This idea is substantiated by several factors: (i) shared characteristics between RCs and leucocytes, including their marginal location in blood vessels [35,42], (ii) the aggregation of RCs at sites of micro- and macroparasite infections [35,36], (iii) close associations between RCs and other fish immune cells, such as mast cells, neutrophils, and epithelioid cells [34], (iv) the presence of immune-active peptides in RCs, including inducible nitric oxide synthase, lysozyme, TNFα, and piscidin 1 [39,43,44], and the presence of Toll-like receptor-2 [45].

RCs have been shown to respond to stressful stimuli, such as parasitic infections and exposure to pollutants. Responses include secretion of their content, recruitment/proliferation, and migration [36,46], with a general consensus that RCs are secretory cells [34,38,39,47,48]. However, the debate regarding their mode of discharge/exocytosis remains open, with some researchers proposing holocrine secretion, releasing the entire content, and leaving an empty capsule, while others suggest apocrine or merocrine modes [35,49,50,51]. The precise mechanisms by which RCs expel their contents are not yet fully elucidated.

Several studies have reported increased abundance and ultrastructural alterations of RCs in different organs of fish exposed to inorganic and/or organic contaminants, including heavy metals and herbicides [17,52,53,54,55,56,57,58]. Recent research by Manera et al. (2022) noted higher RC numbers in the renal hematopoietic tissue of carp exposed to PFOA compared to control fish, along with more frequent RC clusters and a close association between RCs and myeloid lineage cells [17]. These findings suggest the potential immunomodulatory effects of PFOA and form the basis for the present investigation.

This study aims to comprehensively investigate the effects of PFOA on RCs in carp renal hematopoietic tissue, utilising biometry to assess their distribution pattern and electron microscopy to discern ultrastructural evidence of exocytosis across experimental groups. Experimental groups include control fish and fish exposed to environmentally relevant and elevated concentrations of PFOA. The results indicate that RCs are sensitive to PFOA exposure and respond quantitatively by increasing their number and qualitatively by showing ultrastructural signs of enhanced exocytosis. This study contributes to expanding our knowledge on the immunotoxicity of PFOA and on the nature and function of RCs as actors of cellular innate immunity and as promising biomarkers of both exposure and effect.

## 2. Materials and Methods

### 2.1. Fish and Experimental Design

The kidney samples were obtained from a previous study documented in Giari et al. (2016) [59]. A total of thirty-one common carp (adults, two years old; mean total length ± SD, 19.32 ± 2.49 cm; mean weight ± SD, 104.84 ± 27.80 g) were procured from a local fish farm (Azienda Ittica Ferioli, Cento, Ferrara, Italy). Throughout both the four-week acclimation period and the exposure test, the fish were kept under a photoperiod of 14/10 h light/dark and fed manually with a commercial pellet food (Tetra Pond Pellets Mini, Tetra, Melle, Germany) three times per week.

The carp were divided into three groups: an unexposed control group (*n* = 10), and two groups exposed to 200 ng L^−1^ PFOA (PFOA standard, chemical purity 96%, Sigma-Aldrich catalogue number 171468, Merck KGaA, Darmstadt, Germany) (*n* = 10) and 2 mg L^−1^ (*n* = 11) concentrations, respectively, and placed into three 120 L glass aquaria filled with tap water and supplied with a continuous flow of fresh water (at a rate of 500 mL min^−1^). Within each experimental group, a nearly even distribution of sexes, with a ratio approaching 1:1, was ensured. The two treatment aquaria received stock solutions of PFOA dispensed continuously through a peristaltic pump (open system) to maintain the desired concentrations. The 200 ng L^−1^ dose was chosen to represent an environmentally relevant concentration based on PFOA reports in surface water [60], while the 2 mg L^−1^ dose was selected based on data from experimental exposures that elicited a histological response in cyprinid fish [61]. Water temperature, pH, and oxygen saturation in each aquarium were measured three times a week and were maintained at 10–15 °C, 6.70–8.00, and >80%, respectively. After a sub-chronic exposure of 56 days, the carp were euthanised using tricaine methanesulfonate (MS-222) anaesthesia followed by severing the spinal cord and performing necropsies.

Tissue PFOA concentrations in fish exposed to the lowest concentration (200 ng L^−1^) were found to be below the limit of detection (LOD = 0.4 ng g^−1^) using high-performance liquid chromatography with electro-spray ionisation tandem mass spectrometry. However, in fish exposed to the highest tested concentration (2 mg L^−1^), PFOA concentrations were measured at 64.87 ± 24.25 ng g^−1^ in the blood and 1.08 ± 0.54 ng g^−1^ in the kidney (wet weight, mean ± standard deviation) [59]. Further details about the experimental design, fish biometry, and analytical quantification of PFOA concentrations in tissues/organs can be found in Giari et al. (2016) [59].

### 2.2. Light and Transmission Electron Microscopy

To specifically address the present study’s topic, a total of 30 representative kidney samples from 30 carp (10 unexposed, 10 exposed to 200 ng L^−1^ of PFOA, and 10 exposed to 2 mg L^−1^ of PFOA) were collected and processed for electron microscopy as follows: The samples were fixed in 2.5% glutaraldehyde buffered with sodium cacodylate (pH 7.3) at 4 °C for 3 h, post-fixed in 1% osmium tetroxide for 2 h, dehydrated in a graded series of acetone, embedded in epoxy resin (Durcupan™ ACM, Fluka, Sigma-Aldrich, St. Louis, MO, USA), and cut with a Reichert Om U2 ultramicrotome (Reichert-Jung Co., Heidelburg, Germany). Sections of kidney fragments of 1.5 µm thickness (semithin) were cut and then stained with toluidine blue. These stained sections were subsequently examined and photographed using a Nikon Eclipse 80i light microscope (Nikon, Tokyo, Japan) equipped with a digital camera. Ultrathin sections (90 nm) were contrasted with uranyl acetate and lead citrate, examined, and photographed under a Talos L120C transmission electron microscope (Thermo-Fisher Scientific, Waltham, MA, US) operating at 120 kV.

### 2.3. Biometry

For the purpose of this research, only rodlet cells (RCs) within the hematopoietic tissue were recorded, following the methodology described by Manera et al. (2022) [17]. To perform the biometry of RCs using light microscopy at 400× magnification, a single representative semithin section was obtained from each of the five carps in every exposure group, resulting in a total of five semithin sections for each exposure group. Image analysis software (Nis Elements AR 3.0, Nikon, Tokyo, Japan) was employed for this purpose. In each microscopic field screened (measuring 64,000 µm^2^), a total of 29 fields were evaluated in each section, amounting to approximately 1.86 mm^2^ of renal tissue being assessed. To ensure replicability and avoid biases, each tissue section was examined in a zig-zag trajectory, and precautions were taken to prevent duplicate RC counts, account for empty spaces, and avoid analysing non-homogeneous tissue. Moreover, the assessment was carried out without prior knowledge of the exposure group to maintain objectivity.

### 2.4. Statistics

RCs’ distribution throughout the hematopoietic tissue was tested using discrete distribution models, and their frequency per microscopic field was examined for significant differences among experimental groups using the non-parametric repeated measure Friedman test. The statistical analysis was conducted using JASP (version 0.17.3, JASP Team) and JAMOVI (version 2.4.4, The jamovi project) as statistical packages, respectively.

## 3. Results

The following remarks pertain to what is universally regarded as the fully developed state of RC (i.e., mature RC), as, until now, immature forms have not been extensively studied and lack reliable immunohistochemical and/or biomolecular techniques for their unambiguous identification [34,35,39,47,62]. Nonetheless, a putative immature form of RCs is described.

### 3.1. Light Microscopy

**Figure 1:**
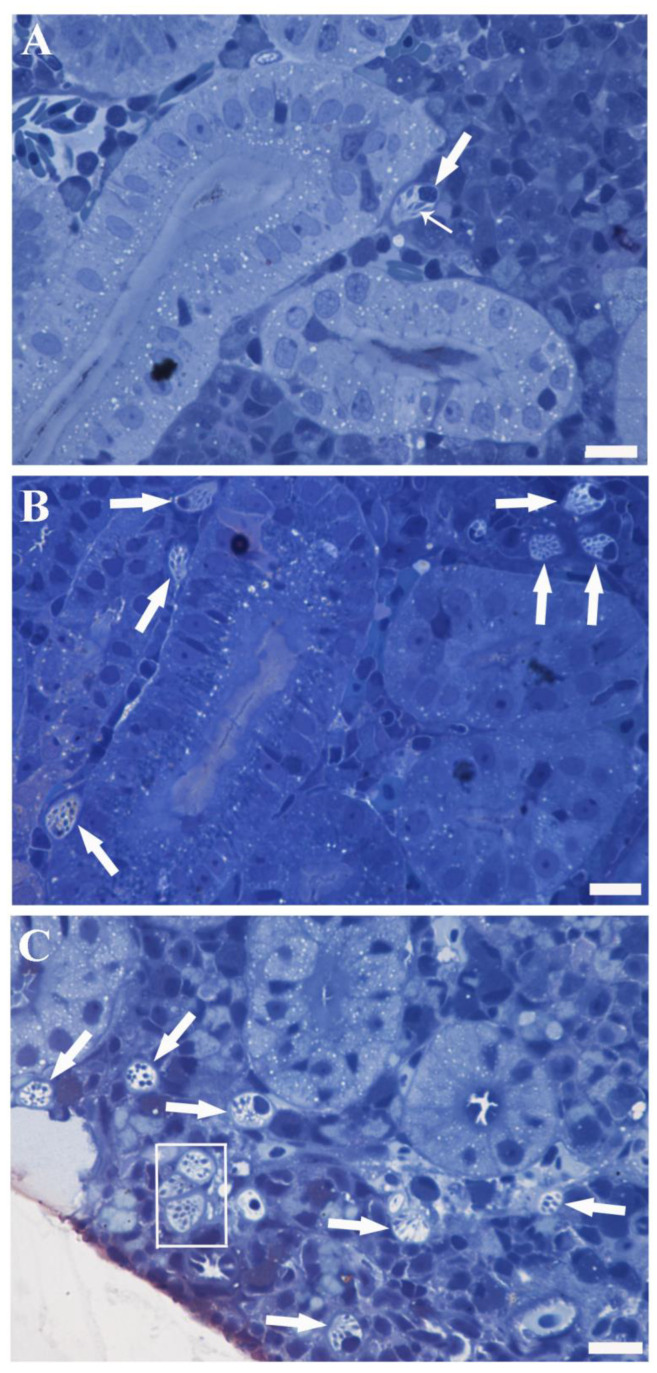
Semithin sections of carp kidneys: unexposed (**A**), exposed to 200 ng L^−1^ PFOA (**B**), and exposed to 2 mg L^−1^ PFOA (**C**). Toluidine blue. Scale bar = 10 µm. (**A**) A solitary RC (thick arrow) is conspicuously present within the hematopoietic tissue, situated between two renal tubules. The distinctive pear-shaped, polarised morphology is clearly discernible, accompanied by the presence of rodlets (thin arrow), vesiculated cytoplasm, and peripheral cytoplasmic thickening. (**B**,**C**) A significant increase in the occurrence of RCs (arrows) is evident, particularly in (**C**), where a cluster of RCs (rectangle) is visible and they appear more translucent and vesiculated.

Although RCs might be found in various locations within the mesonephros (lining blood vessels, in tubules, and collecting ducts), the most prevalent site of occurrence was the hematopoietic interstitium scattered among nephron elements (Figure 1A). Under light microscopy, the typical RC appeared as a pear-shaped polarised cell with a large nucleus at one end of its main axis (referred to as the basal pole) and containing well-known club-shaped granules (rodlets) with their pointed and elongated ends converging towards the opposite cell end (referred to as the apical pole) (Figure 1A). A peripheral cytoplasmic thickening, creating the false impression of a “capsule”, was commonly visible under light microscopy (Figure 1A). This “capsule” was thinner or not observable at the end where rodlets converged (Figure 1A) and could be released into the interstitium (see the ultrastructural section for details). The rest of the cytoplasm appeared clear and vesiculated (Figure 1A). PFOA, especially at a concentration of 2 mg L^−1^, was demonstrated to enhance the occurrence of RCs per single microscopic field, forming clusters (Figure 1B,C). In PFOA-exposed fish, RCs appeared clearer and more vesiculated, with increased evidence of degranulating/degranulated RCs compared to unexposed fish (see the ultrastructural section for details) (Figure 1B).

### 3.2. Biometry

**Figure 2:**
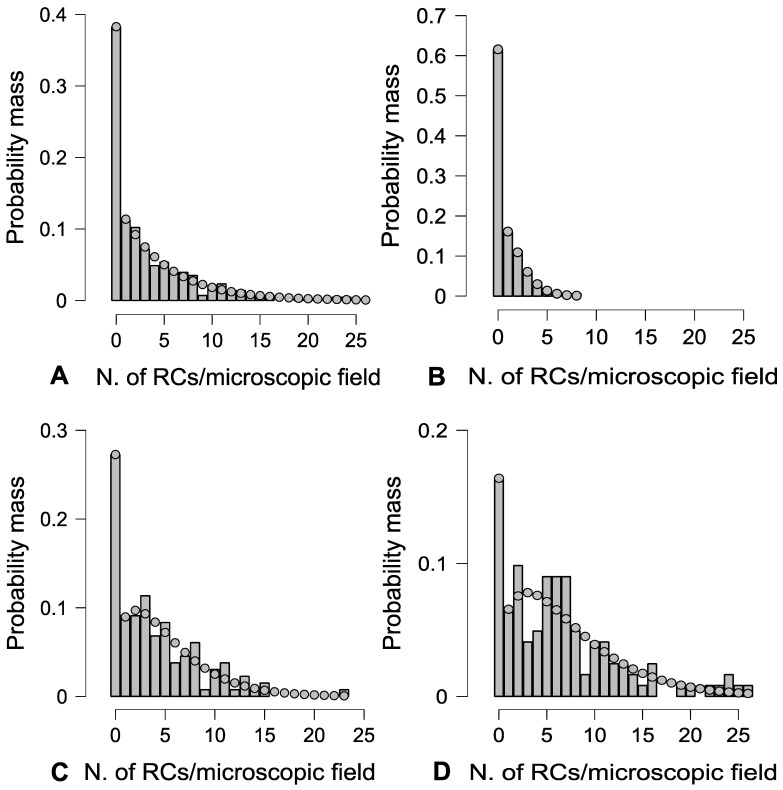
Histogram vs. theoretical (dotted line) probability mass function (according to the zero-inflated negative binomial model) of RC occurrence per microscopic field, on average, irrespective of experimental group (**A**), in unexposed fish (**B**), and in fish exposed to 200 ng L^−1^ (**C**), and 2 mg L^−1^ PFOA (**D**).

RCs were dispersed throughout the hematopoietic tissue based on the zero-inflated negative binomial model in all experimental groups (Figure 2). The frequency distribution of these cells per microscopic field was significantly influenced by PFOA exposure, as evidenced by the Friedman test with a significance level of *p* < 0.01. Specifically, PFOA treatment raised the likelihood of encountering more than a single RC per microscopic field, reaching up to 26 RCs per individual microscopic field at a concentration of 2 mg L^−1^ PFOA (Table 1 and Table 2).

### 3.3. Ultrastructure

**Figure 3:**
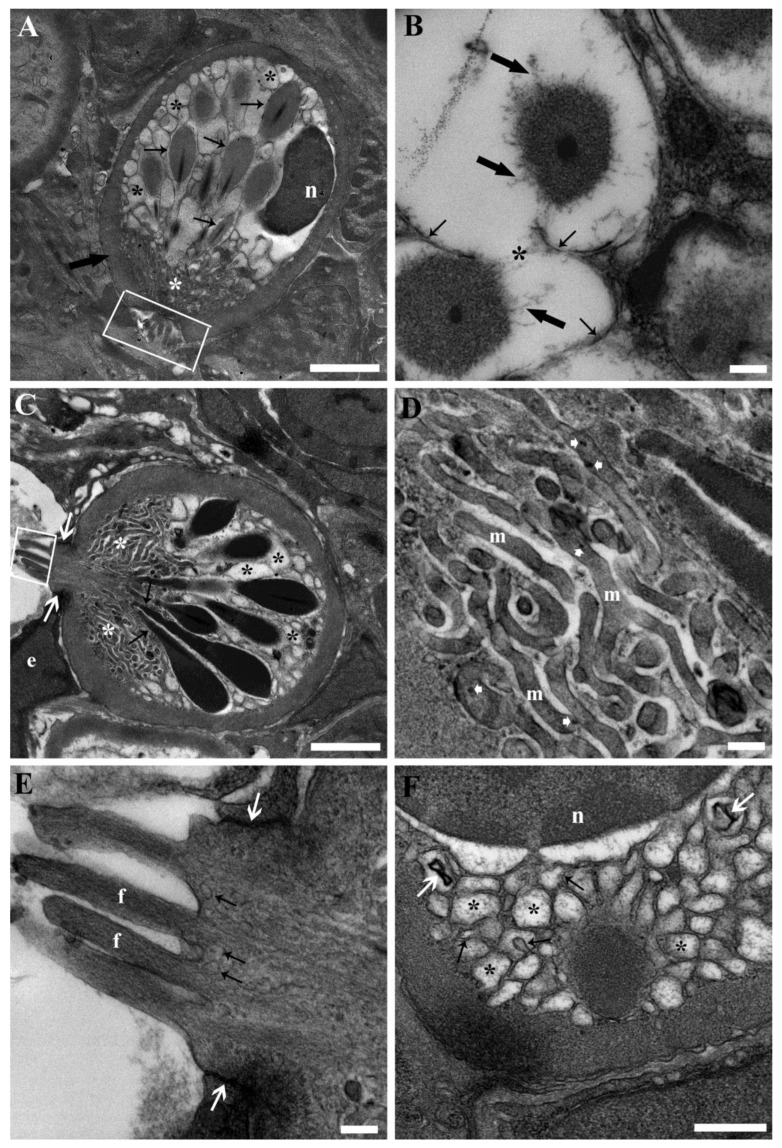
Transmission electron micrographs of RCs from the kidneys of unexposed carp. (**A**) A mature RC exhibits distinctive ultrastructural features, including a pear-shaped, polarised morphology with an oval nucleus (n) at the basal pole. It possesses a fibrillar sub-plasmalemma layer (thick arrow). The RC also contains significantly vesicular cytoplasm (black asterisks) and characteristic rod-like granules (rodlets) (thin arrows) with an amorphous external rodlet sac and an electron-dense core converging at the apical pole. Extremely thin, elongated, and branched mitochondria are particularly abundant in the apical region (white asterisk), where villous-like projections emerge (rectangle). Scale bar = 2 µm. (**B**) The fusion of membranes between adjacent rodlets is discernible (asterisk) in some RCs, accompanied by the dissolution of rodlet sacs (thick arrows) and membrane detachment (thin arrows). Scale bar = 200 nm. (**C**) A RC approaching a sinusoid and extending into its lumen with villous projections (rectangle) is observable, alongside the presence of junctional complexes (white arrows) with the endothelial cells (e) lining the sinusoid. Additionally, a discernible network of mitochondria is evident at the apex of the cell (white asterisks), while the convergence of rodlets (arrows) toward the apex and the residual vesiculated cytoplasm (black asterisks) are also noticeable. Scale bar = 2 µm. (**D**) Detailing the apical network of mitochondria (m), it is noteworthy for its distinctive elongated, thin, and branched appearance, as well as the presence of characteristic calcium granules (arrow heads). Scale bar = 200 nm. (**E**) Detailing the crown-shaped, villous-like projections with a fibrillar core (f) extending into the upper section of the cell, accompanied by vesicles (arrows) at their base. The junctional complexes (white arrows) between the RC and the endothelial cells are appreciable. Scale bar = 200 nm. (**F**) In the cytoplasmic region surrounding the nucleus (n), numerous vesiculations (asterisks) are prominent, with some notable examples of protrusions (black arrows) into adjacent vesicles. Additionally, lamellar bodies are readily discernible (white arrows). Scale bar = 500 nm.

RCs exhibited the characteristic, well-known primary ultrastructural attributes of their developed phase: a polarised, pear-shaped morphology featuring a substantial circular to oval nucleus at the basal pole; a fibrillar sub-plasmalemma layer, corresponding to the “capsule” noticeable under light microscopy; significantly vesicular cytoplasm; distinctive rod-like granules (rodlets) showcasing an amorphous external rodlet sac, and an electron-dense core gathering at the apical pole where the fibrillar sub-plasmalemma layer lessened, eventually favouring the expulsion of the entire cell content (Figure 3A,C).

Cell polarisation was also evident in the cytoplasmic ultrastructure. The rodlets were primarily situated between the nucleus and the apical pole. They were enclosed by a single membrane and occasionally displayed localised rarefaction of rodlet sacs, as well as fusion of membranes among neighbouring rodlets. During such instances, the rodlet membrane detached from the dissolving rodlet sac beneath (Figure 3A,B). The segment of the rodlet sac facing the apical pole could display a coarsely granular appearance. The remaining section of this cytoplasmic portion was occupied by clear, foamy vesicles (Figure 3A,C). Occasionally, these vesicles could protrude into an adjacent vesicle, leading to fusion or the formation of lamellar bodies (Figure 3F). The area of the cytoplasm surrounding and beneath the nucleus was largely filled with the aforementioned vesicles (Figure 3A,C,F). Nonetheless, in certain cases, a shrunken Golgi complex and endoplasmic reticulum Golgi intermediate compartment (ERGIC) were noticeable. The apical cytoplasmic region was primarily occupied by the upper part of the rodlets (specifically their cores), as well as clear, small vesicles and a distinct network of mitochondria (Figure 3A,C). This mitochondrial network was comprised of extremely thin, elongated, and branched mitochondria (Figure 3D). At the apex of the cell, where the sub-plasmalemma fibrillar layer was interrupted, a crown-shaped, villous-like projection could emerge (Figure 3A,C). Within these villous projections, a fibrillar core was evident, extending into the upper section of the cell (Figure 3E). Furthermore, small vesicles were noticeable at the base of the projections, and fusion with the plasmalemma took place (Figure 3E). In some instances, when RCs protruded into blood vessels, junctional complexes with endothelial cells were appreciable (Figure 3C,E). At times, complete expulsion of cytoplasmic content occurred. This led to the discernibility of both rodlets and vesicles within interstitial spaces and/or blood vessels. The remaining portion of the shrunken, effete RCs exhibited an undulating plasma membrane and an irregular, contracted nucleus. Evident condensation and clumping of chromatin accompanied these features.

**Figure 4:**
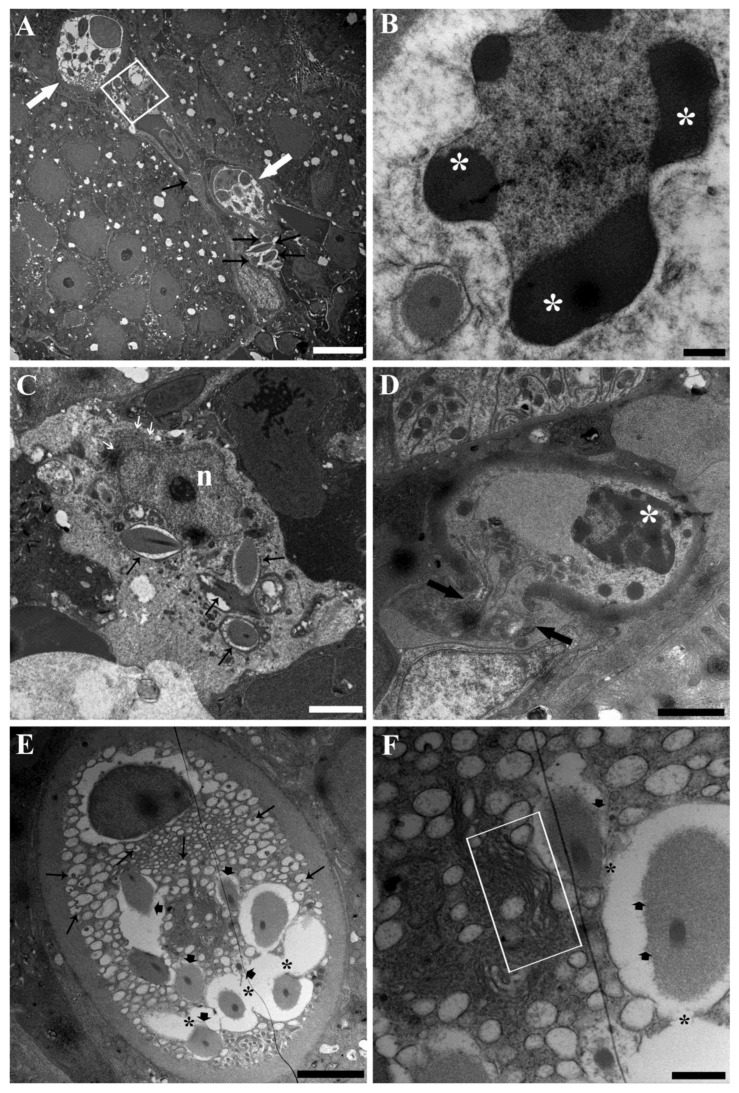
Transmission electron micrographs of RCs from the kidneys of carp exposed to 200 ng L^−1^ PFOA. (**A**) Two RCs displaying enhanced cytoplasmic vesiculation are visible (white arrows), along with an entire discharged RC content (square) and some isolated rodlets (black arrows). Scale bar = 5 µm. (**B**) The nucleus of an effete RC showed condensed, clumped chromatin (asterisks). Scale bar = 500 nm. (**C**) A putative RC immature form is documented, showing an ameboid aspect, not oriented rodlets (black arrows), a large euchromatic nucleus (n) with a prominent nucleolus, and the presence of a rough endoplasmic reticulum (white arrows). Scale bar = 2 µm. (**D**) A RC discharging part of its cytoplasm (arrows) into the hematopoietic interstice is visible, displaying an ondulated, contracted membrane and a nucleus with condensed, clumped chromatin (asterisk). Scale bar = 2 µm. (**E**) A RC shows a microvesiculated cytoplasm with enhanced evidence of vesicles protrusion/fusion within/with adjacent vesicles (arrows). The fusion of contiguous rodlet membranes results in the formation of an intercommunicating channel (asterisks). Rodlet sac dissolution is also visible (arrow heads). Scale bar = 2 µm. (**F**) Detailing the middle part of the cytoplasm shown in (**E**) with a shrunken Golgi complex and ERGIC (rectangle). Rodlet sac dissolution (arrow heads) and rodlet membrane fusion (asterisks) are also appreciable. Scale bar = 500 nm.

**Figure 5:**
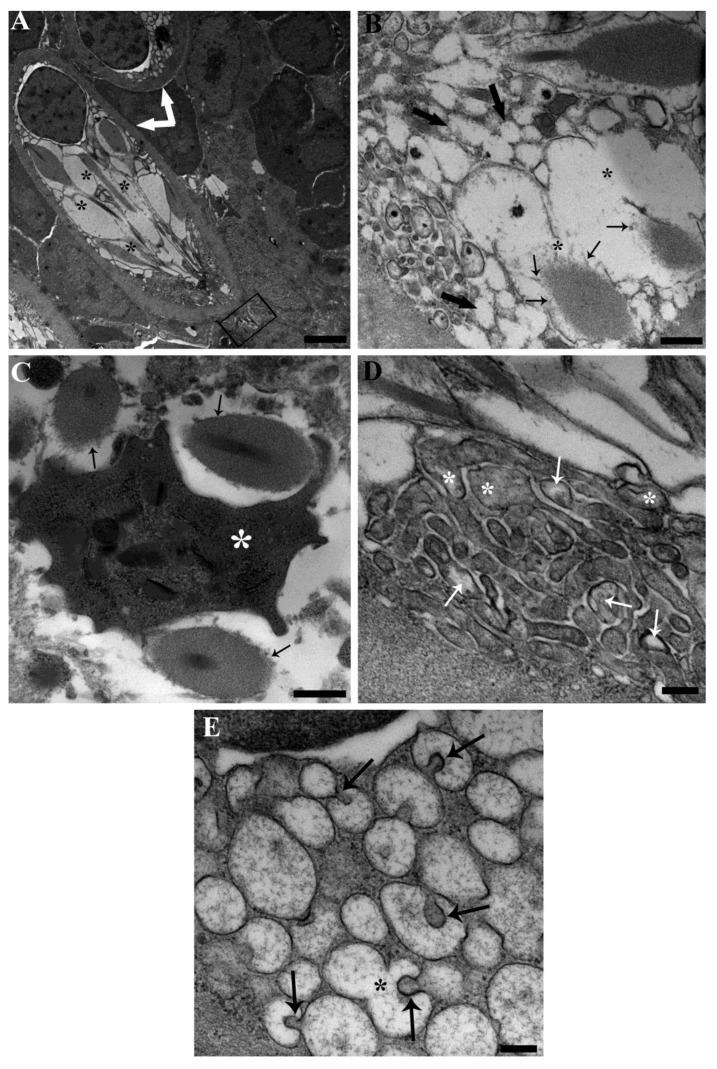
Transmission electron micrographs of RCs from the kidneys of carp exposed to 2 mg L^−1^ PFOA. (**A**) A two-RC cluster is visible (arrows), with an elongated RC showing impressive rodlet sac dissolution (asterisks). Crown-shaped, villous-like projections (rectangle) are appreciable at apex. Scale bar = 2 µm. (**B**) Detailing the enhanced cytoplasmic vesiculation (thick arrows), rodlet membrane fusion (asterisks), and rodlet sac dissolution (thin arrows). Scale bar = 500 nm. (**C**) A neutrophil granulocyte (asterisk) phagocytising three rodlets (arrows) is shown. Scale bar = 500 nm. (**D**) The apical network of mitochondria displays focal swelling (asterisks) and matrix lysis (arrows). Scale bar = 200 nm. (**E**) The vesiculated cytoplasmic portion shows enhanced evidence of vesicles protrusion (arrows) and fusion (asterisk) within/with adjacent vesicles. Scale bar = 200 nm.

PFOA exposure was shown to affect the ultrastructure of RCs (Figure 4 and Figure 5). Specifically, the dissolution of rodlet sacs showed an increased intensity and extent, as did the fusion of rodlet membranes (Figure 4A,E,F and Figure 5A,B). The protrusion and fusion of vesicles within and with adjacent vesicles were significantly heightened (Figure 4E and Figure 5E). All of these changes collectively gave the impression of heightened vesiculation of RCs, noticeable also under light microscopy (Figure 4A and Figure 5A). Some mitochondria displayed focal swelling and matrix lysis (Figure 5D). Moreover, there was an increase in the frequency of complete cytoplasmic expulsion (Figure 4A). Notably, neutrophils were observed phagocytising rodlets (Figure 5C). Additionally, signs of apoptotic nuclear changes were observable in discharged RCs (Figure 4B,D). All the aforementioned alterations in RCs induced by PFOA exposure exhibited a correlation with the concentration to which the fish were subjected. However, there was a noticeable inconsistency observed in fish exposed to 200 ng L^−1^ PFOA. In this group, there appeared to be a higher occurrence of RCs releasing their content completely, accounting for 29% of the total counted RCs. This was in contrast to fish exposed to 2 mg L^−1^ PFOA, where a lower percentage (21%) of RCs displayed this behaviour. In unexposed fish, this phenomenon was observed in only 8% of RCs. It is important to note that the ultrathin sections observed via TEM represented a small tissue area selected through light microscopy, focusing on mature RC occurrences. Identifying single rodlets was challenging through light microscopy. Consequently, the evidence of discharged RCs might have been underestimated, warranting caution when interpreting previous results. Occurrences of putative immature forms were documented in fish subjected to PFOA exposure (Figure 4C). In these instances, sporadically, conspicuous ameboid cells were identified, bearing rodlets, yet devoid of a concurrent fibrillar capsule presence (Figure 4C). These cells exhibited a notable nucleus distinguished by abundant euchromatin content and a prominent nucleolus (Figure 4C). Notably, they were situated in close proximity to cells of the myeloid lineage, specifically neutrophil granulocytes at varying stages of differentiation. Additionally, discernible rough endoplasmic reticulum was observed, coherent with attributes indicative of a metabolically active cellular state (Figure 4C).

## 4. Discussion

The distribution pattern of RCs within tissues has not been subjected to prior, specific statistical analysis, with the notable exception being a prior study involving fish from the current experimental cohort [17]. In that investigation, RCs within the mesonephros were found to adhere to a zero-inflated negative binomial model across all experimental groups, including both unexposed and PFOA-exposed subjects. This suggests a non-random distribution of RCs within mesonephros, in contrast with the distribution expected under a Poisson model. Hematopoietic tissue emerged as the primary location for RCs within the mesonephros [17].

The negative binomial probability distribution, with applications in diverse biological and biomedical scenarios, signifies non-uniform cellular clustering stemming from factors such as cellular attraction, chemotaxis, spatial constraints, variations in cell proliferation rates, irregular migration patterns, or uneven resource distribution within tissues, particularly in situations characterised by overdispersion where variability exceeds that anticipated by a Poisson process [63,64,65,66,67,68,69,70].

In the context of the current findings, it is pertinent to establish a correlation between the observed distribution pattern and the spatial contingencies arising from the renal histoarchitecture, where hematopoietic tissue disperses within the intricate nephron network. Moreover, the distribution of RCs is influenced by genuine contagious and clustering factors. Previous research has suggested that RC degranulation itself could potentially contribute to the recruitment and clustering of these cells [17]. Furthermore, it was postulated that PFOA might enhance this behaviour, possibly through Toll-like receptor (TLR) mediation, as highlighted in the study by Manera et al. (2022) [17]. This proposition gains additional support from investigations into other models, where PFOA’s impact on TLRs has been substantiated [16,71]. Notably, recent work by Alesci et al. (2022) [45] has unveiled that RCs within the kidney of goldfish, a closely related species in comparison to the carp, exhibited immunoreactivity to TLR-2. This finding has prompted speculation that these cells play a pivotal role in the immune response of teleosts [45].

As a result, the documented influence of PFOA exposure on the frequency distribution of RCs within each microscopic field, coupled with an increased likelihood of detecting clusters of RCs and the emergence of potential immature RCs in PFOA-exposed fish, which aligns with the crystalline inclusion phase of the pre-encased stage described by Flood et al. (1975) [62], supports the idea that PFOA exposure contributes to the promotion of RC recruitment.

With regard to the secretion properties of RCs, studies have mainly focused on expulsion of rodlets, possibly since they are their main defining element. Referring to secretion mode, the holocrine modality has been claimed, referring to the abrupt rupture of plasmalemma and discharge of the entire cytoplasm, possibly consecutive to an active contraction of the fibrillar capsule [47,72], though Hawkins (1984) [51] claimed single rodlets may be discharged without plasmalemma rupture in a merocrine way, where the fusion of rodlet membrane with plasmalemma occurs. The apocrine secretion modality has also been suggested by other researchers [48,73].

Characterising rodlet content is relatively underexplored. Leino (1982) [74] revealed positive histochemical responses to carbohydrates and proteins in the rodlet sac, but it did not react to lipid-specific, nucleic acid, or certain carbohydrate stains, such as alcian blue at pH 1.0. Conversely, the granule core positively stained for proteins but not for carbohydrates, nucleic acids, or lipids. Under electron microscopy, the rodlet sac resisted protease digestion and exhibited positive periodic acid-silver methenamine staining, suggesting a prevalence of glycoproteins. In contrast, the inner core, susceptible to protease digestion, did not react with silver methenamine, indicating a relatively pure protein composition [74]. In another study, Iger and Abraham (1997) [52] found that mature RCs in carp and trout contained rodlets with enzymes like alkaline phosphatase (in the rodlet sac) and peroxidase (in the core), as confirmed by immunogold labelling techniques. Bosi et al.’s report (2018) [39] highlighted the immunostain positivity of carp intestinal RCs for the inducible isoform of nitric oxide synthase (i-NOS), lysozyme, and histochemical positivity for lectins ConA, SNA, WGA, and DBA.

Notably, it should be emphasised that nitric oxide, being a gas with an ultrashort half-life, is synthesised on demand and is not stored within vesicles. Given its lipophilic nature, it can freely traverse membranes without necessitating the conventional exocytosis process involving vesicle membrane fusion with the plasmalemma [75]. While light microscopy fails to definitively correlate positivity at the ultrastructural level, the prevailing diffuse and coarse positivity documented in the report of Bosi et al. (2018) [39] appears to encompass the entire cytoplasm, rather than being exclusive to rodlets.

The vesiculations, which are the most prominent features along with rodlets, have been considered dilated, vesiculated, and degranulated endoplasmic reticulum cisternae [47,48,49,76]. Currently, no research suggests that these vesiculations could serve as alternative secretory elements, except for a notable study by Della Salda et al. (1998), which speculates about a subtype of RCs with vesiculations instead of typical rodlets [77]. An alternative interpretation comes from Mattey et al. (1979), who observed RCs with membranous vacuoles, suggesting they might have already secreted their contents without detailing the specific secretory mechanism [78].

Likewise, the notion of rodlets being liberated through mechanisms other than the established abrupt and disruptive process, as well as the prospect of rodlet membrane fusion with the plasmalemma, had not previously been advanced. Cases involving the rarefaction of rodlet sacs, dissolution, and fusion of rodlet membranes, despite being extensively recognised and documented, have primarily been linked to broader alterations [79,80]. These patterns, in fact, are not unique to RCs and have been observed in other cell types (e.g., mast cells), aligning with distinct exocytosis patterns known as “compound exocytosis” and “piecemeal exocytosis”, which notably lack the conventional complete trans-membrane transposition of granule content [81,82]. In piecemeal degranulation, a quiescent secretory granule initially expands and encapsulates a small secretory cargo portion within a budding vesicle. Subsequently, this vesicle detaches, moves towards the plasma membrane, fuses with it, and releases its contents into the extracellular environment [82]. Crivellato et al. (2003) describe piecemeal degranulation as a model for the gradual release of bioactive substances in paracrine and endocrine secretion processes [83]. In compound exocytosis, several secretory granules merge to form a larger one, which then fuses with the plasma membrane, leading to the release of its contents and the emptying of the degranulating sac [82]. In sequential compound exocytosis, a single granule initiates secretion and acts as a conduit for subsequent granules, resulting in the complete discharge of cargo and the emptying of the sac [82].

Considering RCs’ unique structure and potential hindrances to exocytosis due to the sub-plasmalemmal fibrillar capsule, investigating the interactions of piecemeal secretory vesicles and secretory channels with the plasma membrane is crucial. This investigation should focus on the cellular apex, where the fibrillar capsule is absent. Notably, small vesicles have been identified in this region, with indications of increased prevalence following PFOA exposure. Additionally, rodlets have been observed protruding externally from the cell, providing an alternative mode for rodlet content exocytosis [76]. Current findings confirm RCs’ basal exocytosis activity, characterised by piecemeal exocytosis. Intriguingly, exposure to PFOA enhances this basal exocytosis activity in a concentration-dependent manner while promoting increased fusion of the rodlet membrane. This fusion pattern resembles the concept of compound exocytosis.

The mechanistic association between PFOA exposure and the observed upsurge in exocytosis warrants further exploration. It remains to be elucidated whether the increased RC activity, potentially functioning as sentinel cells, in response to PFOA exposure is due to direct stimulation through mechanisms like TLR-mediated pathways, as previously suggested [17,84], indicating a direct causal link between PFOA and RC responsiveness. Alternatively, heightened exocytosis could indirectly result from PFOA exposure, triggered by tissue damage and/or classical sentinel cell activation, suggesting a diagnostic relationship with PFOA. Notably, both PFOA and related compounds can activate and induce degranulation in mammalian mast cells [85,86], potentially involving TLRs among other factors [87,88,89]. PFOA and perfluorooctane sulfonic acid (PFOS) have distinct effects on granule release from mast cells: at low concentrations, they enhance active degranulation machinery, while at higher concentrations, they induce cell lysis [86].

During the well-known disruptive secretion mode, multiple authors have observed pyknotic nuclei in RCs, resembling the nuclear pattern of apoptosis [48,49,76,90]. A crucial distinction between necrosis and apoptosis is their reliance on intracellular ATP levels, as demonstrated by Eguchi et al. (1997) [91]. ATP depletion inhibits Fas/Apo-1-stimulated apoptosis, while ATP replenishment via glycolysis or oxidative phosphorylation reinitiates apoptosis [91]. In contrast, necrosis involves severe mitochondrial dysfunction, rapid energy depletion, and internal homeostasis disruption, as detailed by Leist et al. (1997) [92]. The presence of mitochondria at the apical pole of RCs raises questions about their potential expulsion as initial organelles and their roles in contractile activity and apoptosis. It is conceivable that the contraction phase precedes mitochondrial expulsion, with stored elastic energy in the fibrillar capsule passively released during expulsion, in line with cytoskeletal network mechanics [93]. This underscores the importance of coordinating mechanical dynamics for efficient contractile function. Similarly, the apoptotic cascade should be initiated before explosive discharge. Whether this secretion mode represents regulated exocytosis or a terminal outcome of regulated cell death remains uncertain, necessitating further investigation, including signalling pathways and the potential role of PFOA in their induction.

During this research, neutrophils were observed phagocytising rodlets, resembling the phagocytic activity seen in mammals during anaphylaxis towards mast cell granules [94,95,96,97]. This behaviour is a selective scavenging mechanism that down-regulates actions mediated by granule mediators [95,96,97]. Since rodlets contain enzymes like alkaline phosphatase and peroxidase [52], this process may help mitigate potential tissue damage caused by these enzymes. Additionally, the phagocytosis of rodlets by neutrophils could have other effects, such as potentially influencing the proliferation of the myeloid neutrophilic lineage, as suggested in a previous study [17].

Villous projections, commonly referred to as the “koronenartige Konfiguration” or “crown-shaped configuration” [48,49,98,99,100], exhibit variable occurrence, irregular lengths, and distinctive ultrastructural architecture. They bear structural and potentially functional resemblance to T cell microvilli [101,102], as proposed by Fishelson and Becker in 1999 [103], possibly serving roles in sensing, signalling, and secretion. This underscores the role of RCs in innate cellular immunity, supported by increased RC numbers in infected tissues, their degranulation, and immunoreactivity to lysozyme, inducible nitric oxide synthase, piscidin, and tumour necrosis factor alpha in earlier studies [34,35,38,39,43,44,50]. Previous research has indicated the potential effects of stress hormones and stress-related alterations on RCs [34,50,100]. Furthermore, a unique association between RCs, stromal reticular cells, and myeloid neutrophilic lineage leukocytes was observed in the renal hematopoietic tissue of fish in the current experimental cohort, with elevated levels seen in fish exposed to PFOA [17]. A similar relationship was noted in the bulbus arteriosus of goldfish (*Carassius auratus*), a carp-related species [100], suggesting a microenvironment conducive to RC full development [17,100].

In light of the PFOA concentration-dependent response observed in RCs, including their frequency per microscopic field and subsequent degranulation response, it is noteworthy that this phenomenon was evident even at a PFOA concentration of 200 ng L^−1^. This observation persisted despite the tissue concentration remaining below the LOD (0.4 ng g^−1^). This consistent trend aligns with previous findings, observed not only in tissue-based responses but also in gene expression patterns in fish from the current experimental cohort. Such consistency raises concerns about the reliability of the reference chemical analytical methodology [17,18,24,104,105]. It’s essential to highlight that even if the LOD were lowered to detect even the lowest quantities, detecting PFOA in renal hematopoietic tissue would not reveal discernible distributional, ultrastructural, or functional irregularities in RCs. According to biomarker definitions [106,107], the detection of PFOA in kidney tissue would function, at most, as a biomarker of exposure rather than of effect. In contrast, the PFOA-induced alterations observed in RCs, despite their lack of specificity, serve as a biomarker encompassing both exposure and effect.

Given detection limits and concerns about PFAS toxicity, the U.S. Environmental Protection Agency (EPA) recently issued interim drinking water health advisories for PFOA and PFOS [108]. These advisories specify a PFOA health limit of 0.004 ppt, which is 10^−3^ of the current detection limit. This presents significant challenges in developing ultrasensitive analytical methods [109,110] and highlights global concerns regarding PFAS compounds, particularly PFOA.

## 5. Conclusions

This study explores the morphology, distribution, and function of RCs, revealing their sensitivity to environmental stressors like PFOA. The observed increase in recruitment and exocytosis under PFOA exposure suggests RCs’ potential as sentinel cells responsive to microenvironmental changes, potentially mediated through TLR pathways. RCs consistently respond to low, environmentally relevant concentrations of PFOA, emphasising the need for advanced analytical methods to accurately assess environmental impacts.

In conclusion, this research enhances our comprehensive understanding of RCs, indicating their potential involvement in innate cellular immunity and responses to environmental factors. Their sensitivity to environmental stressors positions them as promising biomarkers for both exposure and effect assessment, particularly in the context of immunotoxicity. Future investigations should focus on elucidating the precise roles of RCs in immune responses and their intricate interactions with environmental factors. 

## Figures and Tables

**Table 1:** Frequency distribution of RCs per microscopic field according to experimental group.

*n*. of RCs Per Microscopic Field	Unexposed	200 ng L^−1^ PFOA	2 mg L^−1^ PFOA
0	89	40	24
1	23	13	10
2	16	13	14
3	9	16	6
4	5	10	7
5	1	12	13
6	1	5	13
7	0	7	13
8	1	9	7
9	0	1	2
10	0	4	6
11	0	5	6
12	0	1	4
13	0	3	4
14	0	1	2
15	0	2	1
16	0	0	4
17	0	0	0
18	0	0	0
19	0	0	1
20	0	0	1
21	0	0	0
22	0	0	1
23	0	1	1
24	0	0	2
25	0	0	1
26	0	0	1

**Table 2:** Pairwise comparison (Durbin—Conover).

	Statistics	*p*
Unexposed vs. 200 ng L^−1^ PFOA	2.40	0.020
Unexposed vs. 2 mg L^−1^ PFOA	4.80	<0.001
200 ng L^−1^ PFOA vs. 2 mg L^−1^ PFOA	2.40	0.020

## Data Availability

All relevant qualitative (figures) and quantitative (table) data is reported in the manuscript.
